# Intervention to improve the appropriate use of polypharmacy for older patients with hip fractures: an observational study

**DOI:** 10.1186/s12877-017-0681-3

**Published:** 2017-12-16

**Authors:** Junpei Komagamine, Kazuhiko Hagane

**Affiliations:** 1 Department of Internal Medicine, National Hospital Organization Tochigi Medical Center, 1-10-37, Nakatomatsuri, Utsunomiya, Tochigi, 3208580 Japan; 2 Department of Pediatric Surgery, National Hospital Organization Tochigi Medical Center, 1-10-37, Nakatomatsuri, Utsunomiya, Tochigi, 3208580 Japan

**Keywords:** Comorbidity, De-prescription, Hip fractures, Polypharmacy, Potentially inappropriate medication

## Abstract

**Background:**

Polypharmacy is frequently observed in hip fracture patients. Although it is associated with an increased risk of hip fracture, polypharmacy often continues after hip fracture recovery. This study aimed to evaluate the effectiveness of an intervention to improve appropriate polypharmacy for elderly patients admitted to the hospital for hip fractures.

**Methods:**

We conducted a retrospective observational study to compare the outcomes of patients receiving the intervention (*n* = 32) with those of patients who received usual care (*n* = 132). All hip fracture patients aged 65 years or older and prescribed 5 or more medications at admission from January 2015 to December 2016 were included in the study. The intervention consisted of an assessment by internal medicine physicians of the appropriateness of polypharmacy and the de-prescription of any unnecessary medications during the patients’ hospital stay. The primary composite outcome was death or the first occurrence of any new fracture. Comparisons between the intervention and usual care groups were analyzed using binary logistic regression.

**Results:**

A total of 164 patients were included in the study. The mean patient age was 84.8 years, and the mean numbers of prescribed medications and potentially inappropriate medications at admission were 8.0 and 1.3, respectively. The mean follow-up period was 8.0 months. The primary composite outcome occurred in 35 (21.3%) patients. The total number of potentially inappropriate medications at discharge was significantly lower in the intervention group than in the usual care group (0.8 ± 0.8 for the intervention group vs 1.1 ± 1.0 for the usual care group; *p* = 0.03). However, no significant differences in the primary composite outcome were found between the intervention and usual care groups (7 in the intervention group and 28 in the usual care group, odds ratio 1.04, 95% CI 0.41–2.65; *p* = 1.00).

**Conclusions:**

The intervention to improve appropriate polypharmacy was associated with a reduction in potentially inappropriate medications but not an improvement in clinical outcomes. This intervention, which focused only on polypharmacy, may not effectively improve outcomes for elderly patients with hip fractures.

**Trial registration:**

UMIN-CTR UMIN000025495. Retrospectively registered 2 January 2017.

**Electronic supplementary material:**

The online version of this article (10.1186/s12877-017-0681-3) contains supplementary material, which is available to authorized users.

## Background

Adverse drug events are a major cause of preventable hospital admissions due to co-morbidities and polypharmacy, particularly among elderly patients [1]. One of the harmful effects of polypharmacy is the increased risk of falls, which can result in hip fractures [2, 3]. Given the burdens associated with mortality and morbidities due to hip fractures [4–6], it is important to avoid polypharmacy among elderly patients.

Nonetheless, polypharmacy is frequent in elderly patients who fall [7] and in hip fracture patients [8]. Furthermore, polypharmacy often continues after hip fracture recovery [7, 8]. Given that older patients who have fallen in the past year are more likely to fall again [9] and that the incidence of a subsequent second hip fracture after the first hip fracture is substantial [10], evaluation and interventions to address polypharmacy in older patients with hip fracture are important. In fact, The American College of Emergency Physicians Geriatric Emergency Department Guidelines recommend a multidisciplinary team intervention for all elderly patients prescribed more than five medications who present to the emergency department, regardless of the presenting complaint [11].

In past decades, several strategies and tools to improve inappropriate medication use in elderly patients have been developed by geriatric experts in several countries [12, 13]. Nonetheless, few studies evaluating the effectiveness of interventions to improve the appropriateness of polypharmacy in elderly hip fracture patients with respect to clinical outcomes such as mortality, cardiovascular events and fractures as the primary aim have been conducted [14–21]. Thus, we investigated whether an intervention to improve the appropriateness of polypharmacy in elderly patients with hip fractures leads to more favorable clinically relevant outcomes.

## Methods

### Study design and participants

This was a retrospective observational study conducted at National Hospital Organization Tochigi Medical Center, a 350-bed acute care hospital in the Tochigi prefecture of Japan. Our hospital had neither geriatricians nor orthogeriatricians. We included all consecutive patients aged 65 years or older who were admitted to the hospital for a hip fracture and who were prescribed five or more medications at admission between January 2015 and December 2016. Eye drops, intranasal infusers, over-the-counter drugs, and topical medications were excluded. As-needed medications were included, although medications that were used for apparent transient disease or symptoms were excluded. Patients with hip fracture due to metastatic bone cancer were excluded. We also excluded patients with a second hip fracture during the study period. Patients who received the polypharmacy intervention by internal medicine physicians were defined as the intervention group and the remaining patients as the usual care group. Our aim was to evaluate whether the polypharmacy intervention improved mortality and re-fracture outcomes among hip fracture patients compared with the outcomes of usual care. Ethical approval for this study was obtained from the local institutional research ethics committee. Data were collected from the medical records of National Hospital Organization Tochigi Medical Center as part of standard patient care. Because this was an observational study without a direct contact with patients, written informed consent was deemed unnecessary. This study was retrospectively registered as UMIN000025495.

### Intervention

Beginning in January 2015, our hospital began screening and intervening to reduce inappropriate medications for elderly patients hospitalized in the orthopedic ward. Pharmacists attempted to contact older patients prescribed five or more medications at admission and their families to inform them of the intervention to improve the appropriate use of polypharmacy. We selected 5 or more medications as the screening criterion based on a previous study [22] because there is no universal standard definition of polypharmacy. The number of medications was determined based on a comprehensive medication history performed by a pharmacist in routine care. For patients whom pharmacists could contact and obtain their written consent to receive the intervention for polypharmacy, the pharmacists made an appointment with one of four internal medicine physicians (including JK) who were interested in polypharmacy among the elderly as soon as possible. The physicians obtained a history from the patients, performed a physical and neurological examination, evaluated the appropriateness of the polypharmacy, changed medications as appropriate and followed up with patients after the consultation until discharge. The appropriateness of medications was determined based on the following: (1) does an evidence-based consensus exist for using the medication for the indication given in this patient; (2) does an indication seem valid and relevant given this patient’s age and disability level; (3) do the known possible adverse reactions of the medication outweigh possible benefits in old, disabled patients; (4) has any harmful event that may be related to the medication occurred; (5) is there another medication or non-pharmacological treatment that may be superior to the current medication. Extrinsic criteria for potentially inappropriate prescription, such as Beers’ criteria [12] and STOPP/START criteria [13], were not used routinely, although all four internal medicine physicians were aware of these criteria. The final decision to stop or change medications was made by internal medicine physicians through shared decision making with patients or their caregivers. Therefore, medications that physicians judged to be inappropriate or unnecessary were sometimes continued due to patients’ preference. For example, patients often refused to stop using or reduce benzodiazepines or hypnotics for insomnia.

### Usual care

Hip fracture patients prescribed five or more medications at admission who could not be contacted by the pharmacist or declined to receive the intervention for polypharmacy were included in the control group. In our hospital, a comprehensive list of current medications was routinely compiled by pharmacists after admission. Pharmacists educated and monitored patients about newly started medications prescribed by the patients’ physician and provided discharge counseling. However, advice by pharmacists about deprescribing and starting medications was not given to the patient’s physician in the absence of apparent harmful effects attributable to the medications. Furthermore, pharmacists neither prepared the summary about discharge medications nor sent it to the primary care physicians and community pharmacists. This is the standard practice for pharmacists in most Japanese hospitals. In our hospital, most hip fracture patients had undergone a surgical procedure by orthopedic surgeons. Orthopedic surgeons also conducted both perioperative and postoperative care for hip fracture patients until discharge. Therefore, medication management during the hospitalization was also performed by orthopedic surgeons. However, medically complicated patients, such as those with insulin dependent diabetes and severe heart failure, consulted with either internists or cardiologists. After discharge, orthopedic physicians routinely follow up with most hip fracture patients until more than six months after discharge.

### Data collection and measurements

#### Data collection

Data were collected using the electronic medical records of National Hospital Organization Tochigi Medical Center. In our hospital, hip fracture patients were routinely followed up by orthopedic surgeons until more than six months after admission. Moreover, our hospital is one of the two largest acute care hospitals, serving approximately 0.5 million individuals in the area around our hospital. Thus, the database of National Hospital Organization Tochigi Medical Center was used for evaluation of the efficacy of the intervention on patient outcomes, such as death or fracture. The last follow-up date was September 11, 2017.

#### Characteristics

Information on age, sex, hip fracture location, past medical history, Charlson Comorbidity Index (CCI) [23], medications, and in-hospital management (surgery, perioperative antibiotic use, venous thrombosis prophylaxis and osteoporosis treatment) was retrieved from the medical records at the time of the patient’s first admission.

#### Outcome measure

The primary composite outcome was death or the first occurrence of any subsequent fracture during the study period. Secondary outcomes included death, any new fractures, cardiovascular events, delirium, adverse drug events, in-hospital infections, or unplanned hospital admission for any reason. We also assessed changes in the number of medications, potentially inappropriate medications (PIMs) and fall-risk-increasing drugs from the time of admission to discharge.

### Definitions

PIMs were defined based on the 2015 Beers Criteria of the American Geriatric Society [12]. Two of the five parts of the Beers Criteria were used: PIM use in older adults and PIM use in older adults due to drug-disease or drug-syndrome interactions that may exacerbate the disease or syndrome. In Japan, few methods to evaluate the appropriateness of medications among older patients have been tested or validated. However, some studies found that the Beers’ criteria might be applicable in a Japanese setting [24, 25], and the Beers’ criteria was used the most frequently in Japanese research. Therefore, we selected the Beers’ criteria. We also defined fall-risk-increasing drugs based on criteria from a previous study [18].

Cardiovascular events included myocardial infarction, heart failure, venous thromboembolism (VTE) and stroke. Adverse drug events were included only if the medication was discontinued due to its harmful effect on the patient. In-hospital infections were defined as any infection requiring antibiotic treatment.

### Statistical analysis

As of December 2016, we, along with other physicians in our hospital, planned to initiate a new strategy including the management of polypharmacy for elderly hip fracture patients after January 2017. Therefore, despite the small sample size, we included hip fracture patients only until December 2016. Furthermore, the rate of hip fracture patients whom pharmacists could not approach was unexpectedly high. This resulted in the small sample size of the intervention group.

The baseline characteristics of the study population were compared using Fisher’s exact test for categorical variables and Student’s t-test for continuous variables. For patients who survived until discharge, the proportion who took PIMs at admission and at discharge was calculated for each group. These proportions at admission and at discharge were then compared for each group using Fisher’s exact test. Clinical outcomes between the intervention and usual care groups were compared using a logistic regression analysis for binary responses and are presented as odds ratios. The last observation carried forward analysis was used for missing data. For primary composite outcome, we also conducted multivariate analysis using binary logistic regression to examine the association between select variables and outcome. The following variables were entered in the logistic regression model: age, sex, CCI, number of medications at admission, number of PIMs at admission, medical consultation, and polypharmacy intervention. In addition, we performed survival analysis for primary composite outcome and its components with the use of the Cox proportional hazard model and the Kaplan-Meier method. Time zero was the day of admission, and observation ended at death, first fracture, or end of observation. Data regarding death up to at least six months was 62.8% complete (shown as Figure S1 in an Additional file 1). All variables are expressed as the mean ± standard deviation (SD) unless otherwise indicated. The analyses were conducted using IBM SPSS Statistics Base version 21.0 (IBM corporation, Nihonbashi, Tokyo, Japan) or Excel statistical software package version 2.11 (Bellcurve for Excel; Social Survey Research Information Co., Ltd., Tokyo, Japan). The level of significance was set at 5%.

## Results

**Fig. 1:**
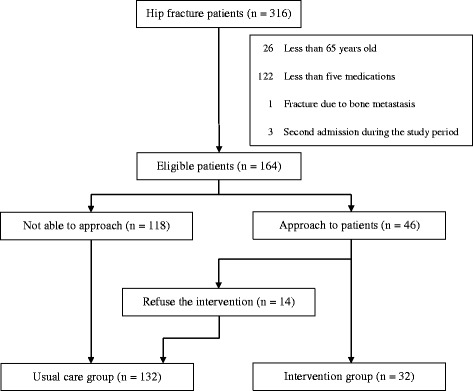
Flowchart of the 164 hip fracture patients

We recorded 316 hip fracture admissions during this period. Among them, 152 admissions did not meet the inclusion criteria (Fig. 1). Overall, 164 patients were included in the final analysis. Of the 164 hip fracture patients, 46 patients were contacted by pharmacists and recommended to receive the intervention for polypharmacy. Of those, 14 patients refused to receive the intervention. In total, 32 patients received the intervention, and 132 patients received usual care. The mean follow-up period was 8.0 months.

The baseline characteristics of the patients are presented in Table 1. For all 164 patients, the mean age was 84.8 years, 35 (21.3%) were male, the mean CCI was 2.3, 69 (42.1%) had dementia, 21 (12.8%) had a previous history of hip fracture, and 54 (32.9%) were institutional residents. The mean numbers of medications, fall-risk-increasing drugs and PIMs at admission were 8.0, 2.7 and 1.3, respectively. Most patients received surgery, perioperative antibiotic treatment and VTE prophylaxis. The baseline characteristics were similar between the two groups, although the intervention group had a higher CCI and number of medications at admission than the usual care group. At admission, the mean numbers of medications, fall-risk-increasing drugs and PIMs were 9.2, 2.9, and 1.5 in the intervention group and 7.8, 2.6, and 1.3 in the usual care group, respectively. At discharge, the mean numbers of medications, fall-risk-increasing drugs and PIMs were 4.5, 1.4, and 0.8 in the intervention group and 6.8, 2.1, and 1.1 in the usual care group, respectively. The total number of fall-risk-increasing drugs and PIMs at discharge was significantly lower in the intervention group than in the usual care group (1.4 ± 1.2 for the intervention group vs 2.1 ± 1.4 for the usual care group; *p* = 0.01; and 0.8 ± 0.8 for the intervention group vs 1.1 ± 1.0 for the usual care group; *p* = 0.03, respectively).

**Table 1:** Characteristics of 164 elderly patients with hip fracture

Characteristics	Intervention *N* = 32	Usual care *N* = 132	*P*-value
Patient characteristics			
Age, mean ± SD	83.8 ± 6.2	85.1 ± 6.7	0.28
Men, n (%)	7 (21.9)	28 (21.2)	1.00
Institutional resident, n (%)	7 (21.9)	47 (35.6)	0.15
CCI, mean ± SD	2.7 ± 1.6	2.0 ± 1.8	0.04
Number of medications at admission			
Total, mean ± SD	9.2 ± 2.5	7.8 ± 2.5	0.01
5–8 medications, n (%)	15 (46.9)	88 (66.7)	0.04
9–12 medications, n (%)	13 (40.6)	38 (28.8)	0.21
13 or more medications, n (%)	4 (12.5)	6 (4.5)	0.11
Number of medications at discharge			
Total, mean ± SD	4.5 ± 2.6	6.8 ± 2.4	< 0.001
0–4 medications, n (%)	18 (56.3)	21 (15.9)	< 0.001
5–8 medications, n (%)	13 (40.6)	85 (64.4)	0.02
9–12 medications, n (%)	0 (0.0)	23 (17.4)	0.02
13 or more medications, n (%)	1 (3.1)	3 (2.3)	1.00
Fall-risk-increasing drugs^a^ at admission			
Total number, mean ± SD	2.9 ± 1.3	2.6 ± 1.4	0.22
0–2 medications, n (%)	13 (40.6)	62 (47.0)	0.56
3–5 medications, n (%)	16 (50.0)	65 (49.2)	1.00
6 or more medications, n (%)	1 (3.1)	5 (3.8)	1.00
Fall-risk-increasing drugs^a^ at discharge			
Total number, mean ± SD	1.4 ± 1.2	2.1 ± 1.4	0.01
0–2 medications, n (%)	27 (84.4)	80 (60.6)	0.01
3–5 medications, n (%)	5 (15.6)	50 (37.9)	0.02
6 or more medications, n (%)	0 (0.0)	2 (1.5)	1.00
PIMs^b^ at admission			
Total number, mean ± SD	1.5 ± 0.8	1.3 ± 1.1	0.19
0–1 medication, n (%)	17 (53.1)	83 (62.9)	0.32
2–3 medications, n (%)	15 (46.9)	44 (33.3)	0.22
4 or more medications, n (%)	0 (0.0)	5 (3.8)	0.58
PIMs^b^ at discharge			
Total number, mean ± SD	0.8 ± 0.8	1.1 ± 1.0	0.03
0–1 medication, n (%)	27 (84.4)	89 (67.4)	0.08
2–3 medications, n (%)	5 (15.6)	41 (31.1)	0.12
4 or more medications, n (%)	0 (0.0)	2 (1.5)	1.00
Past medical history, n (%)			
Dementia	14 (43.8)	55 (41.7)	0.84
Heart disease^c^	5 (15.6)	28 (21.2)	0.63
Stroke	11 (34.4)	28 (21.2)	0.16
Diabetes mellitus	6 (18.6)	35 (26.5)	0.50
Hip fracture	2 (6.3)	19 (14.4)	0.37
Any fracture	14 (43.8)	44 (33.3)	0.31
Current smoker, n (%)	1 (3.1)	11 (8.3)	0.46
Current drinker, n (%)	3 (9.4)	10 (7.6)	0.72
Type of hip fracture, n (%)			
Intracapsular fracture	17 (53.1)	60 (45.5)	0.55
Intertrochanteric fracture	11 (36.7)	67 (50.8)	0.12
Subtrochanteric fracture	4 (12.5)	4 (3.0)	0.05
Others	0 (0.0)	1 (0.8)	1.00
Management during admission			
Surgery, n (%)	31 (96.9)	129 (97.7)	0.58
VTE prophylaxis, n (%)	32 (100)	130 (98.5)	1.00
Perioperative antibiotics, n (%)	31 (100)	129 (100)	1.00
Medical consultation^d^, n (%)	7 (21.9)	41 (31.7)	0.39
Osteoporosis treatment, n (%)			
PTH analogue	2 (6.3)	2 (1.5)	0.17
Bisphosphonate	3 (9.4)	6 (4.5)	0.38
Vitamin D	3 (9.4)	21 (15.9)	0.42

^a^Fall-risk-increasing drugs included antihypertensives, alpha-adrenoreceptor antagonists, opioids, dopaminergic agents, antipsychotics, anxiolytics, hypnotics and antidepressants

^b^PIMs were defined based on the 2015 American Geriatric Society Beers Criteria

^c^Heart disease included angina, myocardial infarction and heart failure

^d^This category included consultation about medical problems other than polypharmacy

Table 2 presents characteristics of PIMs at admission and at discharge in each group among the 158 hip fracture patients who survived until discharge. The most common PIMs were benzodiazepines, proton-pump inhibitors (PPIs), and hypnotics at both admission and discharge. The proportion of patients who took any PIMs significantly decreased from admission to discharge in the intervention group (93.5% at admission and 51.6% at discharge; *p* < 0.001) but not in the usual care group (74.8% at admission and 68.5% at discharge; *p* = 0.33). However, even in the intervention group, more than 20% of patients at discharge used benzodiazepines, and approximately 13% of patients at discharge exhibited potentially inappropriate use of PPIs.

**Table 2:** Characteristics of PIMs at admission and at discharge among the 158 patients who survived until discharge

Category	Intervention *n* = 31			Usual care *n* = 127		
	At admission	At discharge	*p*-value	At admission	At discharge	*p*-value
Any PIMs,^a^ n (%)	29 (93.5)	16 (51.6)	< 0.001	95 (74.8)	87 (68.5)	0.33
Benzodiazepines, n (%)	11 (35.5)	7 (22.6)		43 (33.9)	37 (29.1)	
PPIs, n (%)	10 (32.3)	4 (12.9)		33 (26.0)	31 (24.4)	
Hypnotics^b^, n (%)	5 (16.1)	2 (6.5)		17 (13.4)	13 (10.2)	
Antipsychotics, n (%)	4 (12.9)	2 (9.8)		12 (9.4)	9 (7.1)	
Anticholinergics, n (%)	2 (6.5)	1 (3.2)		13 (10.2)	12 (9.4)	
H_2_-receptor antagonists^c^, n (%)	1 (3.2)	0 (0.0)		9 (7.1)	8 (6.3)	
Anticonvulsants, n (%)	2 (6.5)	2 (6.5)		6 (4.7)	8 (6.3)	
Opioids, n (%)	2 (6.5)	2 (6.5)		5 (3.9)	3 (2.4)	
SSRIs, n (%)	1 (3.2)	1 (3.2)		4 (3.1)	4 (3.1)	
Others, n (%)	5 (16.1)	1 (3.2)		12 (9.4)	11 (8.7)	

^a^PIMs were defined based on the 2015 American Geriatric Society Beers Criteria

^b^This category included non-benzodiazepine benzodiazepine receptor agonist hypnotics

^c^This category included only H_2_-receptor antagonist use for dementia

**Fig. 2:**
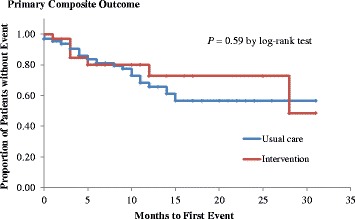
Kaplan-Meier curve for the primary composite outcome event-free survival in the two groups. Plots of time until the primary composite outcome (death or any new fractures) in the intervention group and in the usual care group are shown. The Kaplan-Meier method was used to estimate the cumulative event rate. The log-rank test was used to assess the efficacy of polypharmacy intervention compared with usual care with respect to the primary composite outcome. Data for patients without an event were censored at the time of the last visit

The primary composite outcome occurred in 21.3% of patients (Table 3). Compared with usual care, the intervention was not associated with a reduced risk of the primary composite outcome (odds ratio: 1.04, 95% confidence interval: 0.41–2.65). This result did not change after adjusting for confounding factors at baseline (shown as Table S1 in an Additional file 2). The survival analysis also showed no difference in the event rates of the primary composite outcome between each group (Fig. 2). Among selected variables, CCI was the only variable that predicted the primary composite outcomes. With respect to the secondary outcomes, cardiovascular events and unplanned hospital admissions occurred in 12 (7.3%) and 32 patients (19.5%), respectively. These outcomes also did not significantly differ between the intervention group and usual care group.

**Table 3:** Relevant patient outcome results according to study group

Outcome	Intervention n = 32	Usual care *n* = 132	Odds ratio (95% CI)	*p*-value
Primary composite outcome				
Death or any new fracture, n (%)	7 (21.9)	28 (21.2)	1.04 (0.41–2.65)	1.00
Secondary outcomes				
Death, n (%)	4 (12.5)	13 (9.8)	1.31 (0.40–4.32)	0.75
Any new fracture, n (%)	3 (9.4)	15 (11.4)	0.81 (0.22–2.97)	1.00
Cardiovascular event^a^, n (%)	0 (0.0)	12 (9.1)	Not applicable	0.13
Unplanned hospital admission, n (%)	7 (21.9)	25 (18.9)	1.20 (0.47–3.08)	0.80
Adverse events during hospital stay				
Delirium, n (%)	12 (37.5)	63 (47.7)	0.66 (0.30–1.45)	0.33
In-hospital infection^b^, n (%)	6 (18.8)	17 (12.9)	1.56 (0.56–4.34)	0.38
Adverse drug event^c^, n (%)	1 (3.1)	18 (13.6)	0.20 (0.03–1.59)	0.30
Cardiovascular event, n (%)	0 (0.0)	7 (5.3)	0.26 (0.01–4.63)	0.36
In-hospital death, n (%)	1 (3.1)	5 (3.8)	0.82 (0.09–7.27)	1.00

^a^Cardiovascular events included myocardial infarction, heart failure, venous thromboembolism and stroke

^b^This category included any infections that required antibiotic treatment

^c^Adverse drug events were included only if the medication was discontinued due to its harmful effect

## Discussion

The results of this study demonstrated that the intervention to improve appropriate polypharmacy in elderly patients with hip fracture was associated with a significant reduction in the total number of PIMs but was not significantly associated with a reduced risk of death or any new fracture. The present study was consistent with a previous randomized study [18] in demonstrating that medication reviews do not improve clinically important outcomes among elderly hip fracture patients, although the previous study included hip fracture patients without polypharmacy. Furthermore, our finding was also similar to the results of several meta-analyses showing no efficacy of a medication review on mortality in older patients, although few studies included in these meta-analyses evaluated hip fracture patients [17, 21, 26, 27].

Why does an intervention for polypharmacy in elderly hip fracture patients not improve clinical outcomes? There are several possible reasons. First, given that both acute geriatric unit care [28, 29] and comprehensive geriatric assessment [30] for hospitalized geriatric patients improve patient-relevant outcomes, an intervention focused only on polypharmacy as one of multiple components in geriatric patient care might not be as effective. Second, incomplete interventions to improve the use of inappropriate medications might be the reason that this polypharmacy intervention failed to improve patient-relevant outcomes. In this study, the most common PIMs were sedatives, antipsychotics, PPIs and hypnotics. A substantial proportion of patients in the intervention group used these PIMs at discharge even though sedatives and antipsychotics are associated with an increased risk of falls [31]. Furthermore, the high prevalence of potentially inappropriate use of PPIs at discharge in the intervention group is also problematic because PPI use is associated with an increased risk of hip fracture compared to that in no/past PPI users [32]. These findings were also consistent with those of past intervention studies showing incomplete intervention to improve the appropriateness of medications in a substantial proportion of patients [14, 33]. Third, our intervention focused on inappropriate or unnecessary medications, not potential prescribing omissions (PPOs) [13]. Given that a substantial proportion of hip fracture patients have clinically relevant PPOs [34], a strategy to improve the clinically relevant PPOs might be needed, although it is unclear whether the intervention to improve PPOs results in better patient-relevant outcomes [33]. Fourth, one-time intervention during hospitalization might not be effective, although the efficacy of medication review between in-hospital intervention only and in-hospital intervention with follow-up was not different [35]. These factors might contribute to the negative results in the present study and past intervention studies. Given the high prevalence and harmful effects of polypharmacy among older patients, another effective strategy might be needed.

The interpretation of the results of the present and past studies requires caution as the ineffectiveness of polypharmacy interventions on patient-relevant outcomes does not indicate that medication review for hospitalized geriatric patients is unnecessary. In addition to past studies [16, 17, 21], our findings also suggest that polypharmacy interventions can reduce unnecessary or PIMs safely, although this was not the primary aim of most studies. Considering the cost of medications, regardless of the effect on clinical outcomes, it is important to reduce unnecessary medications safely. Nonetheless, our study and several previous meta-analyses have shown that, although not statistically significant, the odds ratio for death tended to be higher in the medication review group than in the usual care group [17, 26, 27]. Further studies to evaluate the safety of medication review, including deprescribing, among older patients with polypharmacy will be needed.

### Limitations

These results should be interpreted in the context of several limitations. First, our study used a retrospective observational study design, not a randomized study design. Therefore, it is possible that confounding factors influenced our estimates of the association between the intervention and the risk of death or fractures. Furthermore, we used the database of a single center for outcome measures without direct contact with patients. The loss to follow-up rate until six months was also high. These factors might undermine our findings due to uncaptured events. Second, this study was limited to a single center and hospitalized hip fracture patients prescribed five or more medications at admission. Therefore, the results may not be easily generalized to other populations. Third, the 95% confidence interval of the odds ratio for the primary outcome was wide due to the small sample size. Fourth, the follow-up period in this study was relatively short; therefore, the effect on long-term outcomes is unknown. Fifth, our assessment did not include PPOs. Therefore, it was unclear whether our intervention improved PPOs. Sixth, we did not evaluate the change in medications due to a lack of accurate data. Therefore, it is unknown how often withdrawn medications were represcribed after discharge. Seventh, CCI at baseline, which was the only prognostic factor in this study, was higher in the polypharmacy intervention group than in the usual care group. This might underestimate the efficacy of polypharmacy interventions. Finally, our assessment did not include either cost-effectiveness or the effect on the quality of life of elderly patients. Although these limitations are important, given the consistency of our results with those of previous studies and meta-analyses [16, 17, 21, 26, 27], we believe that our findings reflect the true effect of polypharmacy interventions.

## Conclusions

This intervention to improve appropriate polypharmacy in elderly patients with hip fracture was not associated with a reduced risk of death or any new fractures. Interventions focusing on polypharmacy alone might not effectively improve clinically relevant outcomes in elderly patients with hip fracture.

## Additional files

Additional file 1: Figure S1. Flow chart of the participants of the participants until one year after admission. **Figure S2.** Survival analysis of death. **Figure S3** Survival analysis of any new fractures. **Figure S4.** Survival analysis of the primary composite outcome. **Figure S5.** Kaplan-Meier survival curve in the two groups. **Figure S6.** Kaplan-Meier curve for new fracture-free survival in the two groups (DOCX 299 kb)

Additional file 2: Table S1. Summary of logistic regression results to predict the primary composite outcome (DOCX 16 kb)

## Abbreviations

CCI: Charlson comorbidity index
PIM: Potentially inappropriate medication
PPI: Proton-pump inhibitor
PPO: Potential prescribing omission
PTH: Parathyroid hormone
SD: Standard deviation
SSRI: Selective serotonin reuptake inhibitor
VTE: Venous thromboembolism
