# Supplementary material for: Intervention to improve the appropriate use of polypharmacy for older patients with hip fractures: an observational study
Source: BMC Geriatr. 2017 Dec 16;17:288. doi: 10.1186/s12877-017-0681-3 (PMC5732518; doi:10.1186/s12877-017-0681-3)
Supplement: Supplementary file 1 — Flow chart of the participants of the participants until one year after admission. Figure S2. Survival analysis of death. Figure S3 Survival analysis of any new fractures. Figure S4. Survival analysis of the primary composite outcome. Figure S5. Kaplan-Meier survival curve in the two groups. Figure S6. Kaplan-Meier curve for new fracture-free survival in the two groups (DOCX 299 kb) [file 12877_2017_681_MOESM1_ESM.docx]

**Additional file 1 Figure S1.** Flow chart of the participants of the participants until one year after admission.

Intervention group (n = 32))

Usual care group (n = 132)

Death (n = 4)

Loss to follow-up (n = 5)

Death (n = 6)

Loss to follow-up (n = 34)

At 3 months (n = 23)

At 3 months (n = 92)

Death (n = 0)

Loss to follow-up (n = 6)

Death (n = 5)

Loss to follow-up (n = 16)

At 6 months (n = 17)

At 6 months (n = 71)

Death (n = 0)

Loss to follow-up or data missing (n = 5)

Death (n = 2)

Loss to follow-up or data missing (n = 36)

At 12 months (n = 12)

At 12 months (n = 33)

**Figure S2.** Survival analysis of death.

Plots of time until death in the intervention group and in the usual care group are shown. The results were determined based on the basis of Cox proportional-hazards regression analysis. Data for patients without an event were censored at the time of the last visit.

**Figure S3.** Survival analysis of any new fractures.

Plots of time until any new fractures in the intervention group and in the usual care group are shown. The results were determined based on the basis of Cox proportional-hazards regression analysis. Data for patients without an event were censored at the time of the last visit.

**Figure S4.** Survival analysis of the primary composite outcome.

Plots of time until the primary composite outcome (death or any new fractures) in the intervention group and in the usual care group are shown. The results were determined based on the basis of Cox proportional-hazards regression analysis. Data for patients without an event were censored at the time of the last visit.

**Figure S5.** Kaplan-Meier survival curve in the two groups.

*P* = 0.72 by log-rank test

Plots of time until death in the intervention group and in the usual care group are shown. The Kaplan-Meier method was used to estimate the cumulative event rate. The log-rank test was used to assess the efficacy of polypharmacy intervention compared with usual care with respect to death. Data for patients without an event were censored at the time of the last visit.

**Figure S6.** Kaplan-Meier curve for new fracture-free survival in the two groups.

*P* = 0.32 by log-rank test

Plots of time until any new fractures in the intervention group and in the usual care group are shown. The Kaplan-Meier method was used to estimate the cumulative event rate. The log-rank test was used to assess the efficacy of polypharmacy intervention compared with usual care with respect to any new fractures. Data for patients without an event were censored at the time of the last visit.
