# Supplementary material for: Intervention to improve the appropriate use of polypharmacy for older patients with hip fractures: an observational study
Source: BMC Geriatr. 2017 Dec 16;17:288. doi: 10.1186/s12877-017-0681-3 (PMC5732518; doi:10.1186/s12877-017-0681-3)
Supplement: Supplementary file 2 — Summary of logistic regression results to predict the primary composite outcome (DOCX 16 kb) [file 12877_2017_681_MOESM2_ESM.docx]

**Additional file 2 Table S1.** Summary of logistic regression results to predict the primary composite outcome.

| Variables | Odds Ratio (95% CI) | | | |
| --- | --- | --- | --- | --- |
|  | Univariate | *P*-value | Multivariate^a^ | *P*-value |
| Increasing age | 0.98 (0.93-1.04) | 0.55 | 0.98 (0.93-1.04) | 0.60 |
| Male | 1.66 (0.71-3.91) | 0.24 | 1.08 (0.41-2.84) | 0.87 |
| Increasing Charlson Comorbidity Index | 1.29 (1.04-1.61) | 0.02 | 1.30 (1.03-1.65) | 0.03 |
| Increasing number of medications at admission | 1.01 (0.88-1.17) | 0.87 | 0.97 (0.83-1.13) | 0.67 |
| Increasing number of PIMs^b^ at admission | 0.86 (0.60-1.24) | 0.43 | 0.86 (0.59-1.26) | 0.44 |
| Medical consultation^c^ | 1.86 (0.85-4.07) | 0.12 | 1.86 (0.83-4.18) | 0.13 |
| Polypharmacy intervention | 1.04 (0.41-2.65) | 0.93 | 0.98 (0.36-2.66) | 0.97 |

^a^The following variables were adjusted: age, sex, CCI, number of medications at admission, number of PIMs at admission, medical consultation, and polypharmacy intervetion.

^b^PIMs were defined based on the 2015 American Geriatric Society Beers Criteria.

^c^This category included consultation about medical problems other than polypharmacy.
